# Specific tagging of the egress-related osmiophilic bodies in the gametocytes of *Plasmodium falciparum*

**DOI:** 10.1186/1475-2875-11-88

**Published:** 2012-03-27

**Authors:** Anna Rosa Sannella, Anna Olivieri, Lucia Bertuccini, Fabrizio Ferrè, Carlo Severini, Tomasino Pace, Pietro Alano

**Affiliations:** 1 Infectious Diseases Department, Istituto Superiore di Sanità, v.le Regina Elena 299, Rome 00161, Italy; 2 Health Technologies Department, University of Rome "Tor Vergata", Via della Ricerca Scientifica s.n.c, Rome 00133, Italy; 3 Centre for Molecular Bioinformatics (CBM), University of Rome "Tor Vergata", Via della Ricerca Scientifica s.n.c, Rome 00133, Italy

**Keywords:** Malaria, *Plasmodium falciparum*, pfg377, Female gametocyte, Osmiophilic body, Subcellular localization, Gamete egress, Trafficking

## Abstract

**Background:**

Gametocytes, the blood stages responsible for *Plasmodium falciparum *transmission, contain electron dense organelles, traditionally named osmiophilic bodies, that are believed to be involved in gamete egress from the host cell. In order to provide novel tools in the cellular and molecular studies of osmiophilic body biology, a *P. falciparum *transgenic line in which these organelles are specifically marked by a reporter protein was produced and characterized.

**Methodology:**

A *P. falciparum *transgenic line expressing an 80-residue N-terminal fragment of the osmiophilic body protein Pfg377 fused to the reporter protein DsRed, under the control of *pfg377 *upstream and downstream regulatory regions, was produced.

**Results:**

The transgenic fusion protein is expressed at the appropriate time and stage of sexual differentiation and is trafficked to osmiophilic bodies as the endogenous Pfg377 protein. These results indicate that a relatively small N-terminal portion of Pfg377 is sufficient to target the DsRed reporter to the gametocyte osmiophilic bodies.

**Conclusions:**

This is the first identification of a *P. falciparum *aminoacid sequence able to mediate trafficking to such organelles. To fluorescently tag such poorly characterized organelles opens novel avenues in cellular and imaging studies on their biogenesis and on their role in gamete egress.

## Background

Progression of parasite development through diverse intracellular and extracellular forms is accompanied by the biogenesis of several organelles, some of which - rhoptries, dense granules and micronemes - are specific of apicomplexan parasites. Functional role of such organelles is being elucidated in key aspects of parasite development such as invasion of and egress from the host cell, and they are increasingly attracting attention as potential targets of anti-parasitic drugs. Functional studies on protein trafficking to such organelles revealed that specific aminoacid sequences are necessary for proper organelle targeting [1]. Some of such studies, however, indicated that appropriate timing of expression for some of these proteins can be critical for targeting, as shown in the cases of *Plasmodium berghei *protein AMA-1 [2] and *Plasmodium falciparum *proteins RESA and RhopH2 [3,4].

Gametocytes, the blood stages responsible for parasite transmission to the mosquito vector, contain electron dense organelles similar to dense granules, traditionally named osmiophilic bodies [5]. In *P. falciparum *these oval shaped organelles appear on day 4 of sexual differentiation (stage III) [6] and accumulate in female gametocytes, where they progressively increase in number and reach their subcellular localization under the mature gametocyte surface. Ultrastructural observations showed that osmiophilic bodies are connected to the gametocyte surface by ducts and are virtually no longer present after transformation into female gamete, thus supporting the hypothesis of their involvement in gamete egress from the host cell [5,7]. This notion found additional support from functional studies on the *P. falciparum *protein Pfg377, whose ablation by gene knock out caused defective egress of female gametes [8], and the *P. berghei *proteins Mdv-1/Peg3 [9] and PbGEST [10].

In order to provide novel tools in the cellular and molecular studies of the biology of osmiophilic bodies, this work aimed to produce a *P. falciparum *transgenic line, in which a reporter protein fused to a portion of the *pfg377 *coding sequence was specifically targeted to such organelles.

## Methods

### Parasite culture and transfection

The gametocyte-producing *P. falciparum *clone 3D7 was used to derive the transgenic line 3D7/pEpi377. Parasites were cultured and synchronized using standard protocols [11]. For transfection, ring stage parasites (~5% parasitaemia) were electroporated with 80 micrograms of purified plasmid DNA as previously described [12] and drug selected with 2.5 nM WR99210 48 h later. Resistant parasites appeared after approximately three weeks of selection. Gametocyte production was induced by growing parasites to high parasitaemia, while asexual stages were killed by treatment with 0.05 M N-acetylglucosamine for 72 h [13]. Gametocyte activation was induced by exposing mature gametocytes to room temperature in incomplete medium at pH 8.2.

### 5' RACE analysis

5' RACE (Rapid Amplification of cDNA Ends) experiments were performed using the Boehringer Mannheim 5'/3' RACE kit, according to the manufacturer's instructions. Briefly, a first strand cDNA was generated with the *pfg377 *antisense primer pR (see Additional file 1: Table S1 for all primers used), whose sequence is located 30 bp downstream of the gene ATG codon, using 5 micrograms of total mRNA from 3D7 Percoll purified stage III-IV gametocytes. cDNA, purified in GlassMax columns and dCTp/TdT tailed, was used to produce double-stranded DNA by PCR, using the 3' tail specific primer provided by the kit. 5 clones of the resulting PCR products were sequenced.

### Plasmid construction

A 1883 bp fragment, including the putative promoter region and 240 bp of *pfg377 *coding region, was amplified from 3D7 genomic DNA with primers p1 and p2 and cloned into the pHHMC*/3R0.5 plasmid [14], using SacII and XhoI restriction sites. *DsRed *gene was PCR amplified from plasmid pBac(3xP3RED)AgApy [15], with primers p3 and p4 and cloned in the same plasmid with XhoI and KpnI. A second fragment, 385 bp long, spanning the *pfg377 *stop codon was amplified from 3D7 genomic DNA with primers p5 and p6 and cloned downstream the *DsRed *gene using KpnI and NheI restriction sites. Coding sequences were double checked by sequencing, using primers p7 to p9. The plasmid was named pEpi377 and the map is shown in Additional file 2: Figure S1.

### Immunofluorescence analysis

Gametocytes were fixed in 4% paraformaldehyde and blocked in 3% bovine serum albumin overnight, and were simultaneously reacted with the above anti-Pfg377 B portion serum and a polyclonal rabbit anti-DsRed antiserum (MBL International) respectively diluted 1:500 and 1:100. Smears were then incubated with FITC and TRITC-conjugated anti-rat and anti-rabbit IgG, diluted 1:200, and 2 mg/ml DAPI for 1 h. Smears were washed in PBS, mounted and examined on a Leitz DMR fluorescent microscope using an oil immersion objective.

### Immuno electron microscopy

Stage IV and V gametocytes from the 3D7/pEpi377 transgenic line were Percoll purified and processed for immuno-electron microscopy according to published protocols [16]. Briefly, samples were fixed overnight at 4°C with 4% paraformaldehyde/0.1% glutaraldehyde in 0.1 M sodium cacodylate buffer. Next, the suspension was gently washed in sodium cacodylate buffer, dehydrated in ethanol serial dilutions and embedded in LR White, medium-grade acrylic resin (London Resin Company, UK). The samples were polymerized in a 50°C oven for 48 h and ultrathin sections, collected on gold grids, were sunk in 100% ethanol for 3 min, immersed in Tris buffer 0.05 M (pH 10.0) in PCR tubes, and then kept at 99°C for 30 min using a constant temperature box [17].

For immunostaining, the grids were floated on drops of PBS containing 0.1 M glycine for 10 min, washed with PBS, blocked with 5% normal goat serum/1% BSA in PBS for 30 min. For single immunogold-labelling of Ds-Red/Pfg377 fusion protein, ultrathin sections were incubated overnight at 4°C with a 1:50 dilution of rabbit polyclonal anti-DsRed serum (MBL International) in PBS/0.1% BSA/0,05% TWEEN20 buffer. Samples were then rinsed and incubated for 1 h with 10 nm gold-conjugated goat anti-rabbit IgG (SIGMA) (1:50), rinsed again in buffer followed by distilled water and finally air dried. For double immunogold labelling, ultrathin sections were incubated overnight at 4°C with a 1:100 dilution of mouse polyclonal anti-377-B serum [18] in PBS/0.1% BSA/0,05% TWEEN20 buffer. After washing, the grids were incubated for 1 h with 5 nm gold-conjugated goat anti-mouse IgG (Sigma Aldrich) diluted 1:50, rinsed in buffer and further incubated with the above rabbit polyclonal anti-DsRed serum (1:100) overnight at 4°C. Ultrathin sections were then rinsed and incubated for 1 h with 10 nm gold-conjugated goat anti-rabbit IgG (SIGMA) (1:50), rinsed again in buffer followed by distilled water and air dried. Finally, the samples were stained successively with uranyl acetate 2% in H_2_0 and Reynolds lead citrate solution, and observed with an EM208 Philips transmission electron microscope. As controls, the sections were stained without any heating, without the primary antibody and with the diluted gold anti-mouse IgG, or with the diluted mouse pre-immune serum in place of the first antibody.

## Results and discussion

### Regulatory and coding sequences of the *pfg377 *gene for reporter construct

In order to produce a plasmid construct expressing a Pfg377 fragment fused to a reporter protein, for possible targeting to female gametocyte osmiophilic bodies, the following regulatory sequences were used. A 1.6 kb fragment (1643 bp) of genomic region upstream the *pfg377 *ATG codon and a 0.36 kb fragment (361 bp) of downstream region from the gene TAA stop codon were amplified and cloned to drive expression of the *pfg377 *fusion protein. A 5' RACE experiment on total RNA from Percoll purified stage IV gametocytes was preliminarily conducted to investigate length of the 5'UTR. Results showed that all cDNA clones obtained in this experiments extended to nucleotide -294 from the gene start codon, thus indicating that 1.6 kb of upstream sequences was adequate to contain the *pfg377 *promoter.

**Figure 1:**
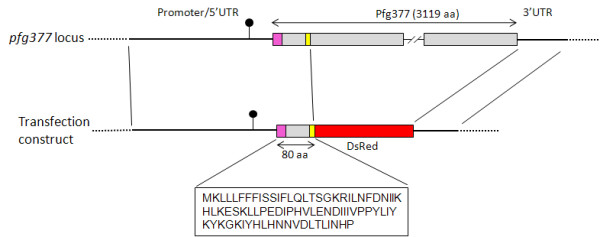
**Diagram of the *pfg377 *genomic regions used in the pEpi377 construct and sequence of the N-terminal portion of the Pfg377 protein fused to the DsRed reporter**. *pfg377 *coding sequence is in grey, signal peptide in pink and the repeat motif in yellow. The position of the furthest nucleotide of the 5*' *RACE cDNA clones is indicated (black lollypop symbol).

As the first 85 amino acids of the *Toxoplasma gondii *rhoptry protein ROP1 and the initial 24 of the *P. falciparum *rhoptry protein RhopH2 were sufficient to drive fluorescent reporters to the respective organelles [4,19], the sequence encoding the first 80 amino acids from *pfg377 *start codon was used in the design of the fusion protein. Next to such 80 aminoacids, a repetitive region encoding a Pro-Glu dipeptide repeat was not included in the chimera. In summary, the above upstream and downstream regulatory regions were cloned flanking a sequence encoding the first 80 aminoacids of Pfg377 fused to the *DsRed *coding sequence (Figure 1). These sequences were cloned in a plasmid containing the *h-dhfr *selection cassette conferring parasite resistance to WR99210, yielding plasmid pEpi377 (Additional file 2: Figure S1).

### Stage-specificity, timing and localization of the transgenic Pfg377-DsRed fusion protein

**Figure 2:**
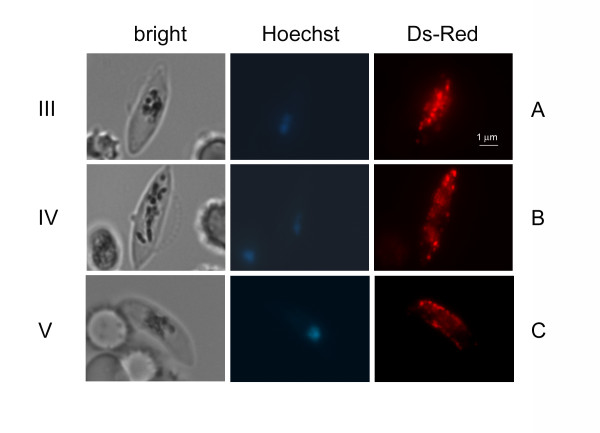
**DsRed fluorescent staining in live female gametocytes of the 3D7/pEpi377 line**. Panels A, B, C: 3D7/pEpi377 female gametocytes respectively at stages III, IV and V of maturation.

The pEpi377 plasmid was transfected in the gametocyte producer clone 3D7 and WR99210 resistant transgenic parasites were selected and analysed. Parasites of the resulting line 3D7/pEpi377 were never seen to fluoresce at any stage of asexual development. A time course of gametocytogenesis in this line was then analysed. Examination of synchronous gametocytes revealed that the round shaped stage I and the crescent shaped stage II gametocytes did not show any fluorescence. A clearly distinguishable fluorescent signal was instead detectable in stage III gametocytes (approximately at day 4 of gametocytogenesis) (Figure 2A), becoming more intense in stage IV and stage V gametocytes (Figure 2B and 2C). Intriguingly, the fraction of gametocytes showing the fluorescent signal was usually around 10%, noticeably lower than an expected 40%. This estimate was based on the fact that several studies using episomal constructs in gametocytes reported that only 50-60% sexual parasites express the episome-encoded fluorescent reporter [20], probably because episomes do not segregate to each daughter cell and gametocytes are not affected by the antifolate WR99210. In addition, the fluorescent signal was predicted to appear only in the fraction of female gametocytes, routinely observed to be 80% in 3D7. A reason for the lower fraction of fluorescent gametocytes observed in the 3D7/pEpi377 line might be that a higher plasmid copy number is required for detection of the Pfg377/DsRed fluorescent signal compared to other fusion proteins. Increasing the concentration of WR99210 to possibly amplify the plasmid copy number however did not result in a higher percent of DsRed fluorescent gametocytes.

**Figure 3:**
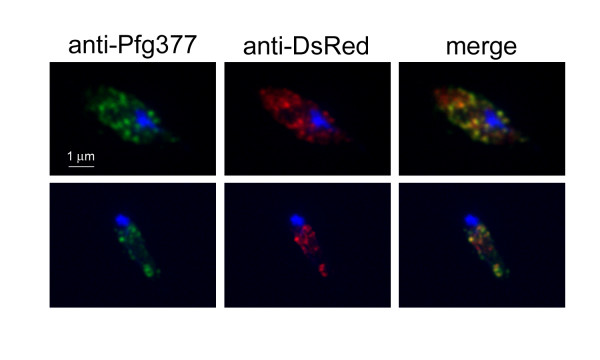
**IFA of 3D7/pEpi377 parasites**. The transgenic Pfg377/DsRed fusion protein, detected by anti-DsRed (red) colocalizes with the endogenous Pfg377 (green) in female gametocytes. Nuclei are stained with DAPI.

Analysis of the pattern of fluorescence in the Pfg377/DsRed-positive gametocytes clearly showed that the fluorescent signal was concentrated in dots evenly distributed in the gametocyte cytoplasm (Figure 2). This feature was reminiscent of the appearance of Pfg377-positive granules in immunofluorescence of female gametocytes, and thus consistent with the possibility that the fluorescence localized to osmiophilic bodies. Immunofluorescence analysis of stage IV gametocytes with antibodies specific for DsRed and for the B portion of Pfg377, which is not included in the Pfg377/DsRed chimera [18], showed that the DsRed-specific signal largely co-localized with that of the anti-Pfg377 antiserum (Figure 3). Partial co-localization is due to the different intensity of the fluorescent signals, being the anti-DsRed signal in general lower, probably due to the lower amount of protein. Finally, as Pfg377 antibodies specifically label female gametocytes [21], the observation that anti-DsRed reactivity was restricted to anti-Pfg377 positive parasites confirmed that the reporter protein is expressed only in female gametocytes.

### Ultrastructural analysis of Pfg377-DsRed localization

**Figure 4:**
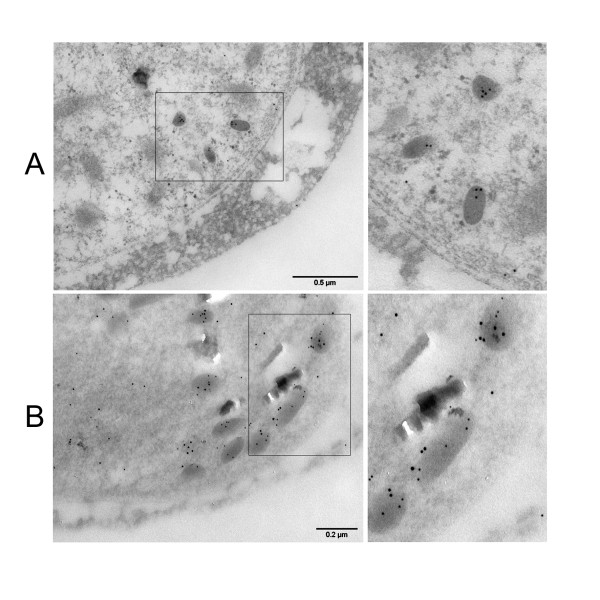
**Immunoelectron microscopy analysis of Pfg377/DsRed fusion protein localization in stage IV female gametocytes**. Panel A: 5 nm gold particles specific for DsRed specifically localize on electron dense osmiophilic bodies. Panel B: 5 nm gold particles specific for the anti-Pfg377 serum and 10 nm gold particles specific for the anti-DsRed antiserum, co-localize on osmiophilic bodies.

In order to unambiguously confirm Pfg377/DsRed co-localization, stage IV and V gametocytes from the 3D7/pEpi377 line were Percoll purified and fixed sections were prepared for immuno-electron microscopy analysis. In one experiment, these were reacted with antibodies against DsRed alone (Figure 4A) and in another one they were simultaneously reacted with antibodies specific for the Pfg377 B portion and for the DsRed moiety of the fusion protein, which were revealed with two secondary antibody-conjugated gold particles of respectively 5 and 10 nm (Figure 4B). Representative images of the reacted gametocyte sections clearly show that the anti-DsRed rabbit antibodies specifically stain osmiophilic bodies (Figure 4A) and analysis of the double immuno-electron microscopy showed that distribution of the different size gold particles almost completely co-localized (Figure 4B). These results in conclusion indicate that the Pfg377/DsRed fusion, after being produced at the appropriate time and stage of sexual differentiation, is trafficked to female gametocyte osmiophilic bodies as the endogenous Pfg377 protein.

### Analysis of the transgenic Pfg377/DsRed fusion protein during gamete emergence

**Figure 5:**
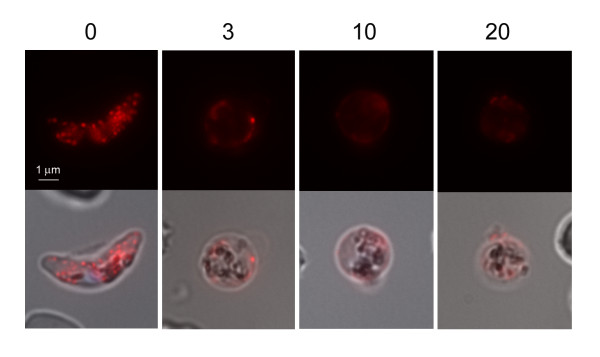
**3D7/pEpi377 live female gametocyte and live gametes at 3, 10 and 20 minutes from triggering gametogenesis**.

As osmiophilic bodies are reported to disappear in female gametes at egress, the expression pattern of the transgenic Pfg377/DsRed fusion protein was analysed during this process. 3D7/epi377 stage V gametocytes were triggered to transform into gametes and the fluorescence pattern was analysed in live parasites collected before and at 3, 10 and 20 minutes post induction. As soon as rounding up was observed the fluorescent dots appeared to be more loosely associated to the female gamete membrane, starting to quiver and gradually disappearing. 10 min after induction, fluorescence was still detectable, although it was weaker than in the elongated gametocyte and its pattern was not granular but diffuse around the cell, progressively fading with time (Figure 5). In conclusion, mobilization and progressive disappearance of Pfg377/DsRed fluorescent dots and their redistribution from an intracellular to an extracellular localization in the transition from stage V female gametocytes to gametes indicates that the fluorescence signal follows the same fate of osmiophilic bodies in female gametogenesis and is consistent with the disappearance of Pfg377 signal in immunofluorescence assays (see Additional file 3: Figure S2). As this process has been so far described only by electron microscopy, this result represents the first dynamic analysis of these events in *P. falciparum *female gametogenesis.

### The N-terminal portion of Pfg377 is sufficient for targeting a fluorescent reporter to female gametocyte osmiophilic bodies

The experiments reported here indicate that the first 80 aminoacids of Pfg377 are sufficient to target the DsRed reporter to female gametocyte osmiophilic bodies. This is to our knowledge the first identification of a *P. falciparum *aminoacid sequence able to mediate trafficking to such organelles, which are comparatively less characterized than similar electron dense organelles such as rhoptries, micronemes and dense granules in other parasite developmental stages. Molecular composition of osmiophilic bodies is also much less characterized than that of the above organelles, as only the gametocyte proteins Pfg377 and PfMdv-1/Peg3, the latter however abundantly present also in additional membrane compartments [22,23], have been so far localized in such organelles in *P. falciparum*. Virtually nothing is known on biogenesis of and protein trafficking to osmiophilic bodies. In general, trafficking to organelles of the apical complex such as rhoptries, dense granules and micronemes have been mainly investigated in asexual stages of *Plasmodium *and *T. gondii*, and requirement of specific sequences in proper organelle localization is still poorly understood. From studies on vesicular trafficking in lysosome biogenesis in higher eukaryotes it results that C-terminal portions of lysosomal proteins are recognized in the Golgi by cytoplasmic adaptor proteins that mediate their trafficking to such organelles. In *T. gondii*, the microneme proteins MIC2 and MIC6 and the rhoptry protein ROP2 reach their organelle destination with such a mechanism [24-26]. At odds with the picture emerging from the above studies, functional analyses of fusion protein localization indicated instead that in *T. gondii *the first 85 aminoacids of the rhoptry protein ROP1 are sufficient to ensure proper organelle targeting [19], and in *P. falciparum *the initial 24 residues of the rhoptry protein RhopH2 are able to drive localization of a fluorescent reporter to such organelles [4]. The work presented here further supports the hypothesis that a signal peptide and a relatively limited N-terminal portion may be sufficient to specifically traffic a parasite protein to the gametocyte osmiophilic bodies.

As only three proteins have been so far positively localized in osmiophilic bodies (Pfg377, Pf Mdv-1/Peg3, PfGEST), a comparative sequence analysis to identify functionally conserved motifs is difficult. Nevertheless, both this approach and structural modeling analysis were undertaken to predict motifs functionally involved in trafficking to such organelles (see Additional file 4). However, the motifs predicted by computational analysis lack in specificity, thus suggesting that a higher number of osmiophilic body-associated proteins is needed to identify the determinants of osmiophilic body localization.

The positive identification presented here of a parasite sequence targeting proteins to female gametocyte osmiophilic bodies is a relevant improvement in the ongoing studies on protein trafficking in parasite sexual differentiation. In addition, the possibility to fluorescently tag such poorly characterized organelles opens novel avenues in cellular and imaging studies on their biogenesis and on their role in gamete egress.

## Competing interests

The authors declare that they have no competing interests.

## Authors' contributions

ARS carried out the molecular and cell biology experiments and participated in the study design. AO and PA conceived the study, participated in its coordination and drafted the manuscript. LB carried out the electron microscopy experiments. FF carried out the bioinformatic analysis. CS carried out the 5' race experiments. TP participated in the design of the study and helped to generate the plasmid. All authors read and approved the final manuscript.

## Supplementary Material

Additional file 1 **Table S1**. Primers used for plasmid construction and sequencing. Click here for file

Additional file 2 **Figure S1**. Map of the pEpi377 plasmid. Click here for file

Additional file 3 **Figure S2**. Immunofluorescence assay with anti-Pfg377 antibodies on a gametocyte culture 10 min after induction of gametogenesis. Parasites were fixed in 4% paraformaldehyde and permeabilized with 1% Triton X-100. Nuclei were stained with DAPI. Images A and B were collected on the same smear with the same exposure time. A. Residual mature gametocyte. B. Female gamete. Click here for file

Additional file 4 **Identification of sequence motifs shared by osmiophilic body-associated proteins **[27-33]. Click here for file
