# Supplementary material for: Specific tagging of the egress-related osmiophilic bodies in the gametocytes of Plasmodium falciparum
Source: Malar J. 2012 Mar 27;11:88. doi: 10.1186/1475-2875-11-88 (PMC3342164; doi:10.1186/1475-2875-11-88)
Supplement: Additional file 1 — Table S1. Primers used for plasmid construction and sequencing. [file 1475-2875-11-88-S1.PDF]

Sannella, Olivieri et al. Additional file 1

Table S1. Primers used for plasmid construction and sequencing.

pR	5’-CCTTTTCCCAGAGGTCAGCTGAAGGAAGATAGACG-3’
p1	5’-GAGCCGCGGTAAGACATTGTAAAATGGCAG-3’
p2	5’-GAGCTCGAGAGGATGGTTTATCAAAGTAAGG-3’
p3	5’-GAGCTCGAG ATGGTGCGCTCCTCCAAGAACG-3’
p4	5’-GGGGTACCCTACAGGAACAGGTGGTGGCG-3’
p5	5’-GGGGTACCGGTCTAGACCTTTACGAATTATAA-3’
p6	5’-GAGGCTAGCGGAACATGATTCTTCTCCCCC-3’
p7	5’-GTATTTTCCATCAATTCATATCG-3’
p8	5’-AGCCCTCGGGGAAGGACAGC-3’
p9	5’-CGGCTCCAAGGTGTACGTG-3’
