# Supplementary figures and images for: Specific tagging of the egress-related osmiophilic bodies in the gametocytes of Plasmodium falciparum
Source: Malar J. 2012 Mar 27;11:88. doi: 10.1186/1475-2875-11-88 (PMC3342164; doi:10.1186/1475-2875-11-88)

**Figure S1.** Map of the pEpi377 plasmid.

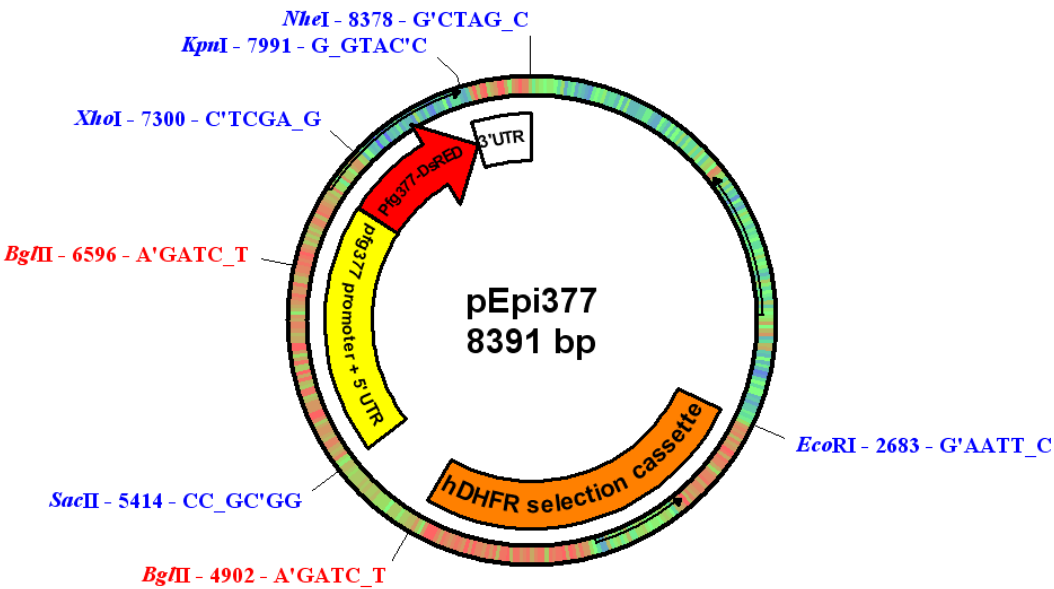

Supplement: Additional file 2 — Figure S1. Map of the pEpi377 plasmid. [file 1475-2875-11-88-S2.PDF]
