# Supplementary material for: Specific tagging of the egress-related osmiophilic bodies in the gametocytes of Plasmodium falciparum
Source: Malar J. 2012 Mar 27;11:88. doi: 10.1186/1475-2875-11-88 (PMC3342164; doi:10.1186/1475-2875-11-88)

Sannella, Olivieri et al. Additional file 3

Figure S2. Immunofluorescence assay with anti-Pfg377 antibodies on a gametocyte culture 10 min after induction of gametogenesis. Parasites were fixed in 4% paraformaldehyde and permeabilized with 1% Triton X-100. Nuclei were stained with DAPI.

Images A and B were collected on the same smear with the same exposure time.

A. Residual mature gametocyte. B. Female gamete.

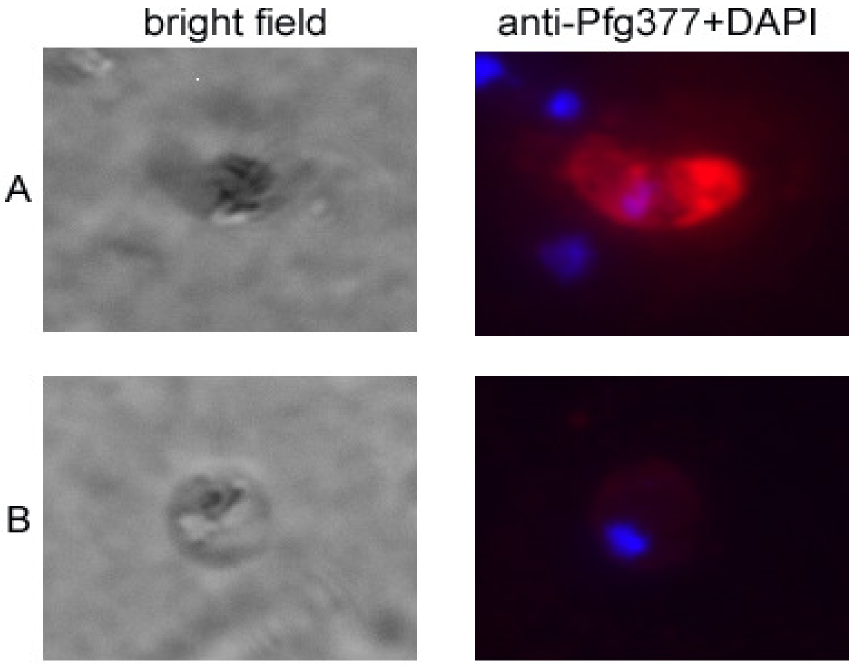

Supplement: Additional file 3 — Figure S2. Immunofluorescence assay with anti-Pfg377 antibodies on a gametocyte culture 10 min after induction of gametogenesis. Parasites were fixed in 4% paraformaldehyde and permeabilized with 1% Triton X-100. Nuclei were stained with DAPI. Images A and B were collected on the same smear with the same exposure time. A. Residual mature gametocyte. B. Female gamete. [file 1475-2875-11-88-S3.PDF]
