# Supplementary material for: Specific tagging of the egress-related osmiophilic bodies in the gametocytes of Plasmodium falciparum
Source: Malar J. 2012 Mar 27;11:88. doi: 10.1186/1475-2875-11-88 (PMC3342164; doi:10.1186/1475-2875-11-88)
Supplement: Additional file 4 — Identification of sequence motifs shared by osmiophilic body-associated proteins [27-33]. [file 1475-2875-11-88-S4.PDF]

Identification of sequence motifs shared by osmiophilic body-associated proteins.

To our knowledge, only three *P. falciparum* proteins are known to be localized in the osmiophilic bodies: PFL2405c (Pfg377)[1], PFL0795c (Pf MDV-1/peg3, the PbMDV1/PEG3 ortholog)[2] and PF14_0467 (the PbGEST ortholog)[3]. These three proteins seem evolutionary unrelated and are very different in size (PFL2405c: 3.119 amino acids; PFL0795c: 221 amino acids; PF14_0467: 254 amino acids). Psipred-predicted secondary structure[4] suggests a mostly helical fold, with extended coil regions. A 13-residue long helix, overlapping or immediately following the signal peptide, is found in all 3 proteins. A ClustalW multiple alignment of the first 80 residues of PFL2405c with PFL0795c and PF14_0467 full-length coding sequences receives a ClustalW score of 70, and shows very little similarity.

We used the MEME suite[5] to scan the first 80 residues of PFL2405c, PFL0795c, and PF14_0467 for shared short sequence motifs that could be involved in the osmiophilic bodies translocation, and applied a list of criteria to pinpoint the most promising protein candidates. A relatively small number of motifs (6 in total) are identified by MEME as having an e-value ≤ 1 and an individual p-value of occurrence of the motif in the input sequence ≤ 0.001. None of these motifs appears to be highly conserved in the 3 input proteins, being generally clusters of charged, polar or hydrophobic residues. We used FIMO[6] to scan through the *P. falciparum* proteome (5.491 annotated proteins) to find other occurrences of each MEME motif. FIMO identified a grand total of 2753 matches, this large number likely due to the little specificity of most motifs.

In order to prune the number of unspecific matches, (i) we masked low complexity regions in *P. falciparum* proteins, that could lead to a large number of false hits; (ii) we

focused on proteins having a canonical signal peptide, which is a common feature of all three known osmiophilic body-associated proteins.

The presence of vast low complexity regions (LCRs) in several *P. falciparum* proteins[7] can inflate the number of hits of motifs matching repetitive regions. Therefore, such regions were masked while searching for motif matches, using the program segmasker, part of the NCBI BLAST+ suite[8]. We additionally filtered all proteins not having a canonical N-terminal signal peptide as predicted by SignalP[9], requiring that both the SignalP neural network module and the SignalP HMM module agree on the prediction.

The filtering procedures reduced the number of matches to 192 total hits in 140 proteins. The lack of motif specificity suggests that a higher number of known osmiophilic body-associated proteins would be needed to identify more stringent determinants of protein translocation to these organelles. The identified motifs and their total number of matches after each filtering step are reported in the Table below.

Protein sequence motifs identified in osmiophilic body-associated proteins.

Motif	Best match[1]	Total[2]	LCR[3]	SignalP[4]	LCR + SignalP[5]
[FY][ITY][HSY][CS]I	FISCI	473	416	35	30
F[ML][DIQ]L[LT]	FMILL	444	394	49	42
V[DFN][YL][AFT][LP][FIL][NF][YH]	VNYFLINY	515	383	51	35
[SN]F[DEI][DN]I	SFIDI	514	449	36	30
[GLT][KL][LNV][VF][NF]	LKNVN	384	299	30	21
[LMQ][10][HLN][GL][NL]	LSNGN	423	293	41	34
		2753	2234	242	192

1. motif best match based on its PSSM; 2. number of motif hits in the whole proteome; 3. motif hits in the proteome after masking for LCRs; 4. motif hits on proteins carrying a canonical signal peptide as predicted by SignalP; 5. motif hits on LCR- proteins carrying a canonical signal peptide.

References:

1. Alano P, Read D, Bruce M, Aikawa M, Kaido T, Tegoshi T, Bhatti S, Smith DK, Luo C, Hansra S *et al*: **COS cell expression cloning of Pfg377, a *Plasmodium falciparum* gametocyte antigen associated with osmiophilic bodies**. *Mol Biochem Parasitol* 1995, **74**(2):143-156.
2. Ponzi M, Siden-Kiamos I, Bertuccini L, Curra C, Kroeze H, Camarda G, Pace T, Franke-Fayard B, Laurentino EC, Louis C *et al*: **Egress of *Plasmodium berghei* gametes from their host erythrocyte is mediated by the MDV-1/PEG3 protein**. *Cell Microbiol* 2009, **11**(8):1272-1288.
3. Talman AM, Lacroix C, Marques SR, Blagborough AM, Carzaniga R, Menard R, Sinden RE: **PbGEST mediates malaria transmission to both mosquito and vertebrate host**. *Mol Microbiol* 2011, **82**(2):462-474.
4. Bryson K, McGuffin LJ, Marsden RL, Ward JJ, Sodhi JS, Jones DT: **Protein structure prediction servers at University College London**. *Nucleic Acids Res* 2005, **33**(Web Server issue):W36-38.
5. Bailey TL, Elkan C: **Fitting a mixture model by expectation maximization to discover motifs in biopolymers**. *Proc Int Conf Intell Syst Mol Biol* 1994, **2**:28-36.
6. Grant CE, Bailey TL, Noble WS: **FIMO: scanning for occurrences of a given motif**. *Bioinformatics* 2011, **27**(7):1017-1018.
7. Pizzi E, Frontali C: **Low-complexity regions in *Plasmodium falciparum* proteins**. *Genome Res* 2001, **11**(2):218-229.
8. Camacho C, Coulouris G, Avagyan V, Ma N, Papadopoulos J, Bealer K, Madden TL: **BLAST+: architecture and applications**. *BMC Bioinformatics* 2009, **10**:421.
9. Petersen TN, Brunak S, von Heijne G, Nielsen H: **SignalP 4.0: discriminating signal peptides from transmembrane regions**. *Nat Methods* 2011, **8**(10):785-786.
10. Gatei W, Kariuki S, Hawley W, ter Kuile F, Terlouw D, Phillips-Howard P, Nahlen B, Gimnig J, Lindblade K, Walker E *et al*: **Effects of transmission reduction by insecticide-treated bed nets (ITNs) on parasite genetics population structure: I. The genetic diversity of *Plasmodium falciparum* parasites by microsatellite markers in western Kenya**. *Malar J* 2010, **9**:353.
